# Association of interleukin-6 gene polymorphisms with the risk of hepatocellular carcinoma: An up-to-date meta-analysis

**DOI:** 10.1097/MD.0000000000023659

**Published:** 2020-12-11

**Authors:** Pei-Pei An, Li-Na Feng, Xiao-Xue Zhang, Qing-Long Jin

**Affiliations:** a Institute of Translational Medicine; b Department of Hepatology, the First Hospital of Jilin University, Changchun, Jilin province, China.

**Keywords:** hepatocellular carcinoma, interleukin-6, polymorphisms, susceptibility

## Abstract

**Background:**

This study was aimed to evaluate the association between interleukin-6 (IL-6) gene polymorphisms and the risk of hepatocellular carcinoma (HCC) in a meta-analysis.

**Methods:**

A literature search was performed for case-control studies published during May, 1993 to May, 2020 focusing on IL-6 gene polymorphisms (–174G > C, –572G > C, and –597G > A) and HCC susceptibility by using PubMed, Cochrane Database, EMBASE, Web of science, and China National Knowledge Infrastructure. From 128 full-text articles, 11 were included in this meta-analysis. I^2^ index was used to assess heterogeneity and Newcastle-Ottawa Scale was utilized for quality assessment.

**Results:**

For IL-6 –174G > C polymorphism, in codominant (GG vs CC: odds ratios [OR] = 2.78, 95% confidence intervals [CI] = 1.25–6.19, *P* = .01, I^2^ = 16%) and recessive (GG+GC vs CC: OR = 2.76, 95% CI = 1.29–5.90, *P* = .009, I^2^ = 3%) models, IL-6 –174G>C polymorphism was significantly associated with the risk of HCC. In dominant (GG vs CC+GC: OR = 1.80, 95% CI = 0.92–3.54, *P* = .09, I^2^ = 86%) and allele (G vs C: OR = 1.49, 95% CI = 0.95–2.32, *P* = .08, I^2^ = 68%) models, IL-6 –174G>C polymorphism had no impact on the risk of HCC. However, in non-Italian Caucasian population, IL-6 –174G>C polymorphism was significantly related to the occurrence of HCC in both dominant (GG vs CC+GC: OR = 3.26, 95% CI = 2.29–4.65, *P* < .00001, I^2^ = 0%) and allele (G vs C: OR = 2.48, 95% CI = 1.48–4.15, *P* = .0006) models. Such correlations also could be observed when healthy individuals were selected as controls. For IL-6 –572G>C and –597G>A polymorphisms, no significant association was observed in all models, regardless of the source of control and population subgroups. No publication bias could be calculated when Begg and Egger tests were employed.

**Conclusion:**

This meta-analysis indicated that IL-6 –174G>C polymorphism was significantly related with the risk for HCC, especially in non-Italian Caucasian population. No significant association was observed for the correlation between IL-6 –572G>C and –597G>A polymorphisms and HCC susceptibility.

## 1 Introduction

Hepatocellular carcinoma (HCC) is the fourth most common cancer and also is the fourth leading cause of cancer-related death worldwide.^[1,2]^ The risk factors for HCC include cirrhosis, hepatitis B virus (HBV) or hepatitis C virus infection, alcohol addiction, metabolic liver disease, and exposure to dietary toxin such as aflatoxins and aristolochic acid.^[2]^ Recently, multi-omics technologies, such as miRNome^[3]^ and proteome,^[4]^ shed new light on the pathogenesis of HCC, which may be beneficial for the diagnosis, prognosis, and treatment of patients with HCC. Current therapeutic options, such as surgery, chemotherapy, liver transplantation, and radiofrequency ablation, only benefits a small percentage of patients. Immunotherapy is an effective and promising treatment approach for HCC. Many studies seek to evaluate the efficacy of immunotherapy, including immune checkpoint inhibitors,^[5]^ cancer vaccines,^[6]^ and adoptive cell transfer,^[7]^ yielding some encouraging results. However, further studies need to focus on overcoming the resistance of immunotherapy.

A persistent, nonspecific, and ineffective activation of the immune system within the chronically inflamed liver is thought to promote carcinogenesis.^[8]^ For example, in a recent study, Toll-like receptor 3 polymorphisms were reported to be a novel risk factor for hepatitis C virus-related HCC.^[9]^ Furthermore, cirrhosis caused by chronic inflammation was present in approximately 80% to 90% patients with HCC.^[10]^ The primary trigger for inflammation which is related with hepatocarcinogenesis is death of epithelial cells, and subsequently multiple inflammatory pathways (e.g., Interleukin [IL]-6, tumor necrosis factor-α, nuclear factor kappa B) contribute to the inflammation-mediated hepatocarcinogenesis.^[11]^

Interleukin-6 (IL-6) is a multifunctional potent pleiotropic inflammatory cytokine and a major driver of hepatocyte repair and replication, which is also a critical mediator of HCC development.^[10]^ In diethylnitrosamine induced mouse model of HCC, IL-6 was demonstrated to play very critical roles in both malignant transformation of HCC progenitor cells and HCC growth.^[8,12]^ IL-6 can signal through 2 distinct pathways: the IL-6 classic and the IL-6 trans-signaling pathway. Bergmann and colleagues found that only IL-6 trans-signaling is essential to promote HCC development via preventing DNA-damage-induced hepatocyte apoptosis and inducing endothelial cell proliferation to promote tumor angiogenesis.^[13]^ Moreover, in patients with HCC, increased IL-6 levels could be observed.^[14,15]^ Single nucleotide polymorphisms of non-coding promoter sequence in IL-6 gene will impact the expression of IL-6, which was considered to be associated with the susceptibility of HCC. Although a meta-analysis published in 2014 demonstrated that IL-6 –174G>C, not –572G>C, polymorphism was related with HCC susceptibility,^[16]^ controversial results also published thereafter.^[17–19]^ Accordingly, an up-to-date meta-analysis is needed to perform. The aim of this study is to evaluate the association between IL-6 gene polymorphisms and HCC development with more studies and larger participant samples. Furthermore, subgroup analysis may discover potential relationships between IL-6 gene polymorphisms and HCC development, which may be beneficial for basic or clinical research in the future.

## 2 Subjects and methods

### 2.1 Literature search

A systemic literature search was performed by 2 authors independently for studies published during May, 1993 to May, 2020 using PubMed, Cochrane Database, EMBASE, Web of science, and China National Knowledge Infrastructure. The search terms were: “interleukin-6” or “IL-6”, “hepatocellular carcinoma” or “liver cancer” or “hepatocellular cancer”, and “SNPs” or “polymorphism”. The resulting articles were examined and unrelated articles were excluded. Additionally, articles in the reference list were manually searched for potentially relevant studies. When more than one of the same patient population was included in different publications, only the most recent or complete study was used. This study was approved by the ethics committee of the First Hospital of Jilin University.

### 2.2 Inclusion and exclusion criteria

**Figure 1:**
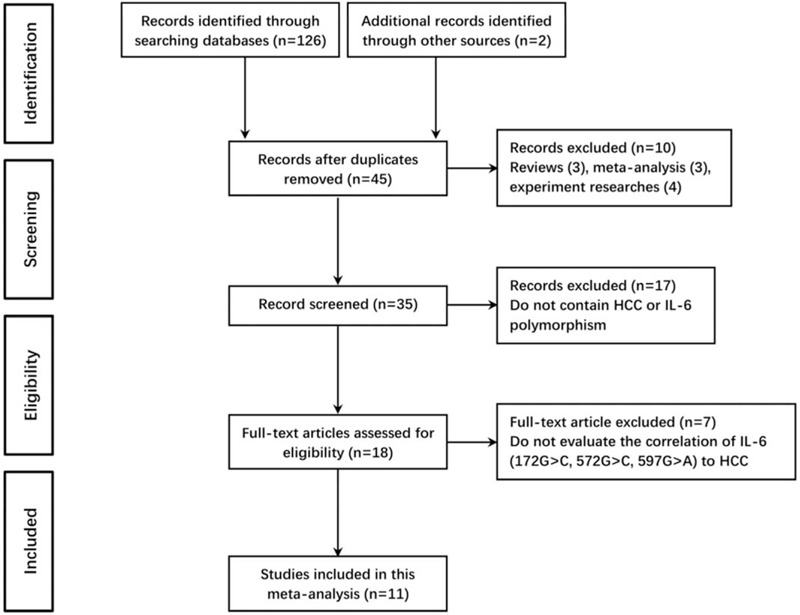
Flow chart of literature selection in this meta-analysis.

Preferred Reporting Items for Systematic Reviews and Meta-Analyses was adopted to report our results. An article was considered relevant if it reported original data from case-control study, regardless of language, investigating the correlations between IL-6 polymorphisms and HCC susceptibility. Controls were healthy individuals, patients with hepatitis B or C virus infection, or patients with hepatitis cirrhosis. The reasons for exclusion from our studies were: studies with insufficient genotyping data of patients or control group; duplicated publication; review articles; experiment researches; studies without control group. If disagreements exist between the 2 reviewers regarding inclusion of a study, consensus was used to resolve such problem. Eleven studies of full-text articles were selected for inclusion in this meta-analysis, and the selection process of studies in this analysis was shown in Figure 1.

### 2.3 Data extraction and quality assessment

The date was extracted by 2 reviewers independently from all eligible studies according to the inclusion and exclusion criteria above, and the quality of each study was assessed by using the Newcastle–Ottawa Scale (NOS), and 9 points represent the highest quality in this scale. Disagreements was resolved by a third reviewer.

### 2.4 Data analysis

Review Manager (RevMan) 5.3 (The Cochrane Collaboration, the Nordic Cochrane Centre, Copenhagen, Denmark.) (Cochrane database) was utilized to analyze the data of included studies. The results were reported as odds ratios (OR) with 95% confidence intervals (CI). Heterogeneity between studies was assessed by using the I^2^ statistic: values of 25%, 50%, and 75% represent mild, moderate, and severe heterogeneity, respectively. Based on results of the heterogeneity test, a fixed effect model was used if *P*>.10, while a random effects model was performed if *P* ≤ .10. Begg and Egger test were employed to evaluate the publication bias across studies with Stata software (version 16.0). (Stata Corporation, College Station, TX, USA) A *P* < .05 was considered statistically significant.

## 3 Results

### 3.1 Characteristics of included studies

The characteristics of included studies were summarized in Table 1. Two studies were from Egypt,^[19,20]^ 2 from China,^[18,21]^ 2 from Italy,^[14,22]^ 1 from Israel,^[23]^ 1 from Korea,^[24]^ 1 form Japan,^[25]^ 1 from India,^[17]^ and the remaining 1 from USA.^[26]^ For –172G>C, –572G>C, –597G>A polymorphisms of IL-6, the number of included studies were 5, 7, and 2, respectively. Eventually, 2013 patients and 3217 control were included in this meta-analysis. All studies had a NOS score ≥7, with an average of 7.36 (Table 1).

**Table 1:** Characteristics of the included studies.

			Number				
Author, yr	Country	Ethnicity	Patients	Control	Source of control	Determination of polymorphism	NOS scores
Ben-Ari et al 2003	Israel	Caucasians	10	125	Healthy control HBV patients	PCR	7
Park et al 2003	Korea	Non-Caucasians	221	475	Liver cirrhosis patients	PCR-RFLP	7
Migita et al 2005	Japan	Non-Caucasians	48	188	HBV patients	PCR	8
Falleti et al 2009	Italy	Caucasians	66	389	Healthy control Liver cirrhosis patients	PCR-RFLP	9
Ognjanovic et al 2009	USA	Caucasians	117	121	Healthy control HBV/HCV patients	Taq Man	8
Giannitrapani et al 2011	Italy	Caucasians	105	193	Healthy control Liver cirrhosis patients	PCR-RFLP	7
Bei et al 2014	China	Non-Caucasians	720	784	Healthy control HBV patients	RT-PCR	7
Saxena et al 2014	India	Caucasians	61	342	Healthy control HBV patients	PCR-RFLP	7
Tang et al 2014	China	Non-Caucasians	505	395	HBV patients	RT-PCR	7
Madkour et al 2017	Egypt	Caucasians	60	105	Healthy control HCV patients	Taq Man	7
Badawy et al 2019	Egypt	Caucasians	100	100	Healthy control	PCR-RFLP	7

NOS = Newcastle-Ottawa Scale, PCR = polymerase chain reaction, PCR-RFLP = PCR restriction fragment length polymorphism, RT-PCR = real-time PCR.

### 3.2 Correlations of IL-6 polymorphisms and HCC susceptibility

#### 3.2.1 For IL-6 –174G>C polymorphism

**Figure 2:**
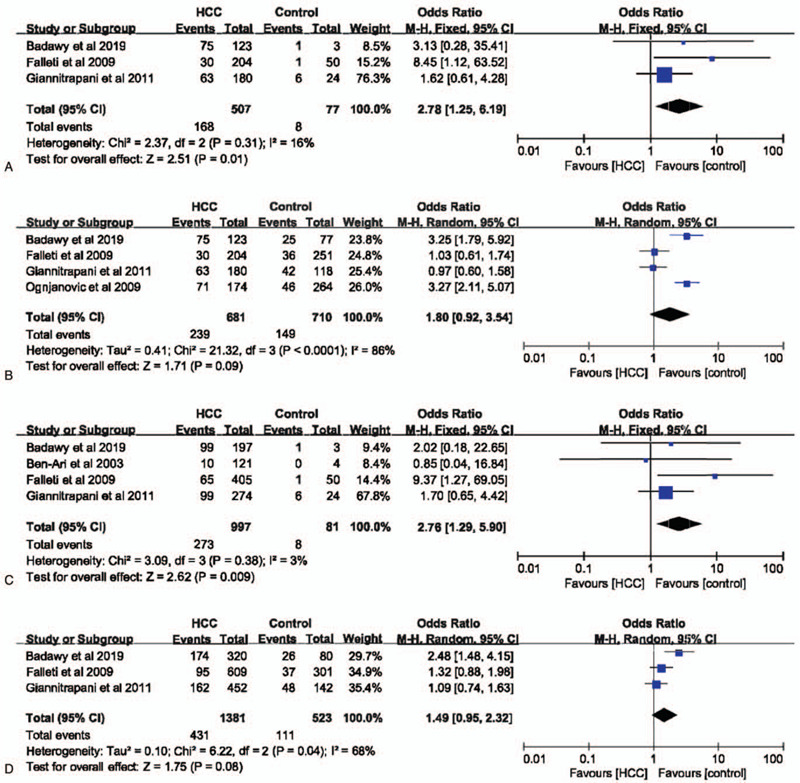
The association between interleukin-6 gene –174G>C polymorphism and hepatocellular carcinoma susceptibility based on overall controls. A. Codominant model; B. Dominant model; C. Recessive model; D. Allele model.

**Figure 3:**
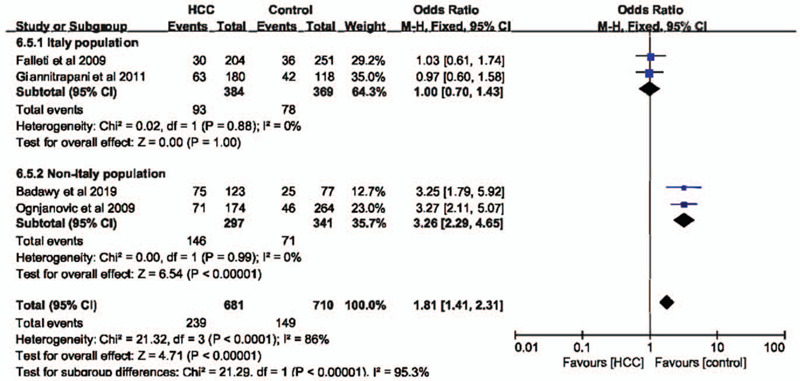
The association between interleukin-6 gene –174G>C polymorphism and hepatocellular carcinoma susceptibility in Italy and non-Italy populations in dominant model based on overall controls.

**Figure 4:**
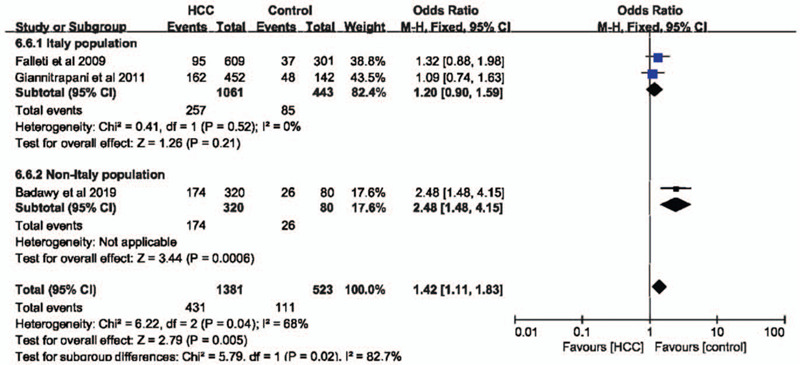
The association between interleukin-6 gene –174G>C polymorphism and hepatocellular carcinoma susceptibility in Italy and non-Italy populations in allele model based on overall controls.

In codominant (GG vs CC: OR = 2.78, 95% CI = 1.25–6.19, *P* = .01, I^2^ = 16%)(Fig. 2A) and recessive (GG+GC vs CC: OR = 2.76, 95% CI = 1.29–5.90, *P* = .009, I^2^ = 3%)(Fig. 2C) models, IL-6 –174G>C polymorphism was significantly associated with the risk of HCC. However, in dominant (GG vs CC+GC: OR = 1.80, 95% CI = 0.92–3.54, *P* = .09, I^2^ = 86%)(Fig. 2B) and allele (G vs C: OR = 1.49, 95% CI = 0.95–2.32, *P* = .08, I^2^ = 68%)(Fig. 2D) models, IL-6 –174G>C polymorphism had no impact on the risk of HCC. Considering the high heterogeneity in these 2 models, a subgroup analysis was performed to evaluate the association between IL-6 –174G>C polymorphisms and HCC susceptibility. Finally, we found that in the non-Italian Caucasian population, IL-6 –174G>C polymorphism was significantly related with the occurrence of HCC in both dominant (Fig. 3) and allele models (Fig. 4) without heterogeneity.

**Figure 5:**
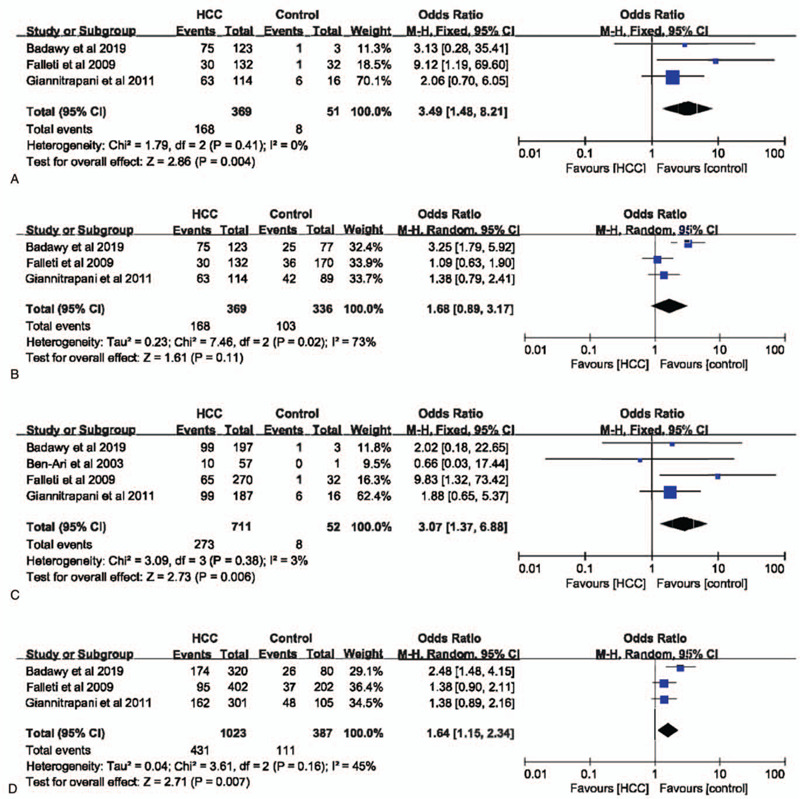
The association between interleukin-6 gene –174G>C polymorphism and hepatocellular carcinoma susceptibility based on normal controls. A. Codominant model; B. Dominant model; C. Recessive model; D. Allele model.

Taken the control group containing patients with hepatitis or cirrhosis into account, we performed a subgroup analysis with healthy individuals as controls. In codominant (GG vs CC: OR = 3.49, 95% CI = 1.48–8.21, *P* = .004, I^2^ = 0%), recessive (GG+GC vs CC: OR = 3.07, 95% CI = 1.37–6.88, *P* = .006, I^2^ = 3%), and allele (G vs C: OR = 1.64, 95% CI = 1.15–2.34, *P* = .007, I^2^ = 45%) models, IL-6 –174G>C polymorphism was significantly associated with the risk of HCC, which was absent in dominant model (GG vs CC+GC: OR = 1.68, 95% CI = 0.89–3.17, *P* = .11, I^2^ = 73%) (Fig. 5). Similarly, in non-Italian Caucasian population subgroup, –174G>C polymorphism of IL-6 gene was significantly related with HCC incidence in dominant model (supplemental Fig. 1).

#### 3.2.2 For IL-6 –572G>C polymorphism

**Figure 6:**
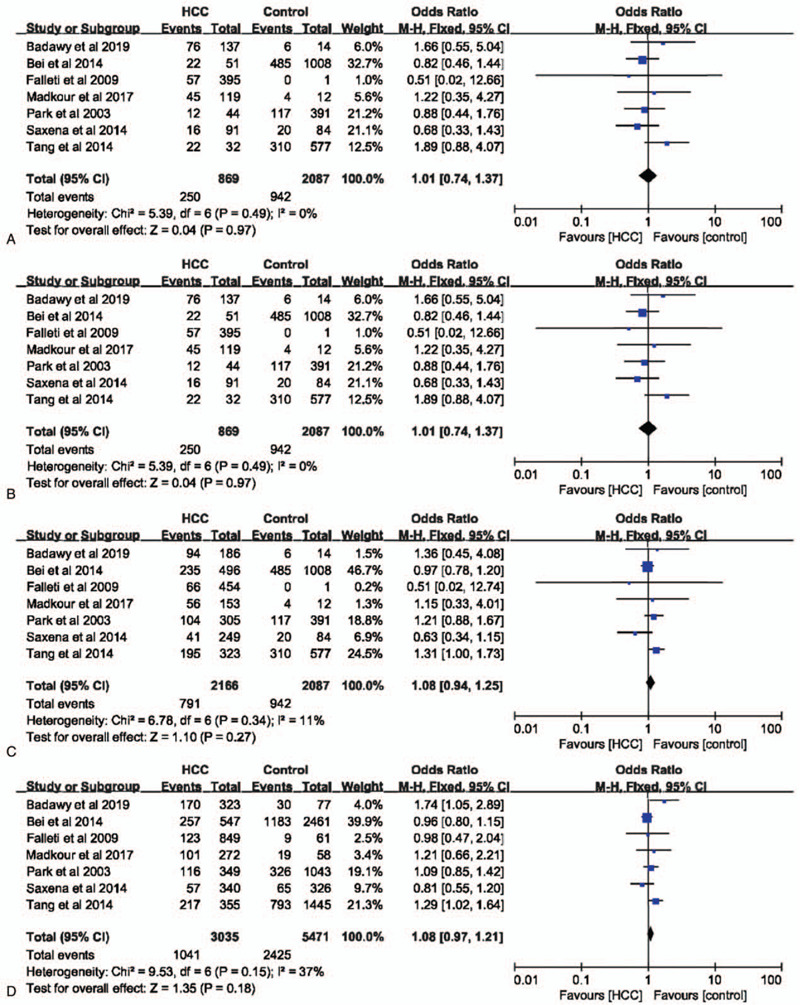
The association between interleukin-6 gene –572G>C polymorphism and hepatocellular carcinoma susceptibility based on overall controls. A. Codominant model; B. Dominant model; C. Recessive model; D. Allele model.

**Figure 7:**
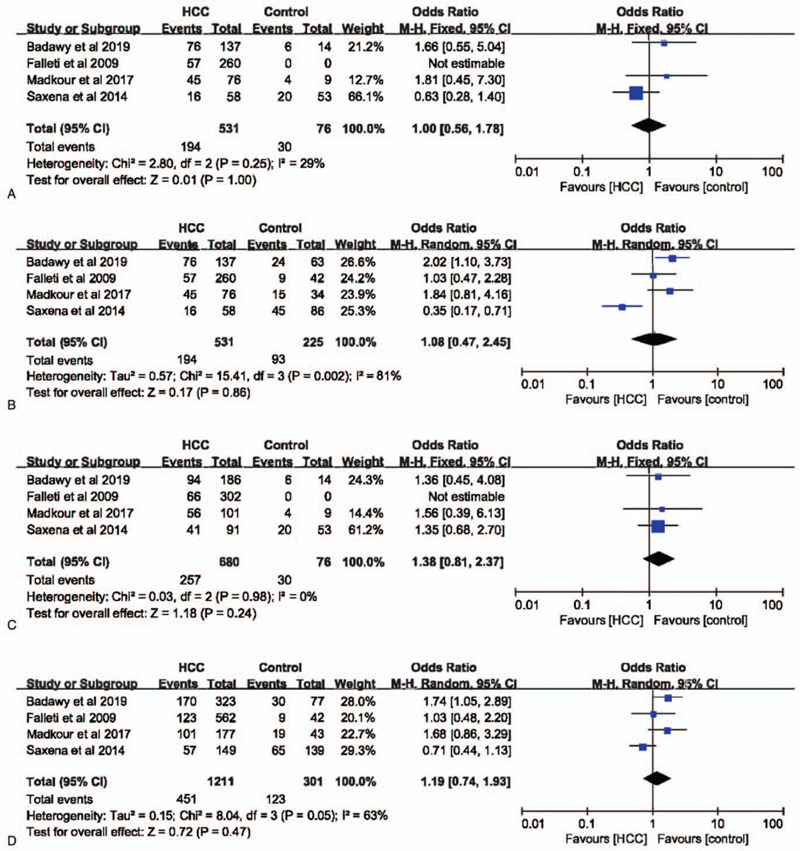
The association between interleukin-6 gene –572G>C polymorphism and hepatocellular carcinoma susceptibility based on normal controls. A. Codominant model; B. Dominant model; C. Recessive model; D. Allele model.

IL-6 –572G>C polymorphism was not significantly associated with the risk of HCC in dominant (GG vs CC+GC: OR = 1.13, 95% CI = 0.89–1.45, *P* = .32, I^2^ = 22%), recessive (GG+GC vs CC: OR = 1.08, 95% CI = 0.94–1.25, *P* = .27, I^2^ = 11%), allele (G vs C: OR = 1.08, 95% CI = 0.97–1.21, *P* = .18, I^2^ = 37%), and codominant (GG vs CC: OR = 1.01, 95% CI = 0.74–1.37, *P* = .97, I^2^ = 0%) models (Fig. 6). In a subgroup analysis with healthy individuals as controls, IL-6 –572G>C polymorphism had no influence on the risk of HCC in all models as well (Fig. 7). Furthermore, in both Caucasians and non-Caucasians populations, IL-6 –572G>C polymorphism had no impact on HCC susceptibility (supplemental Figs. 2–5).

#### 3.2.3 For IL-6 –597G>A polymorphism

**Figure 8:**
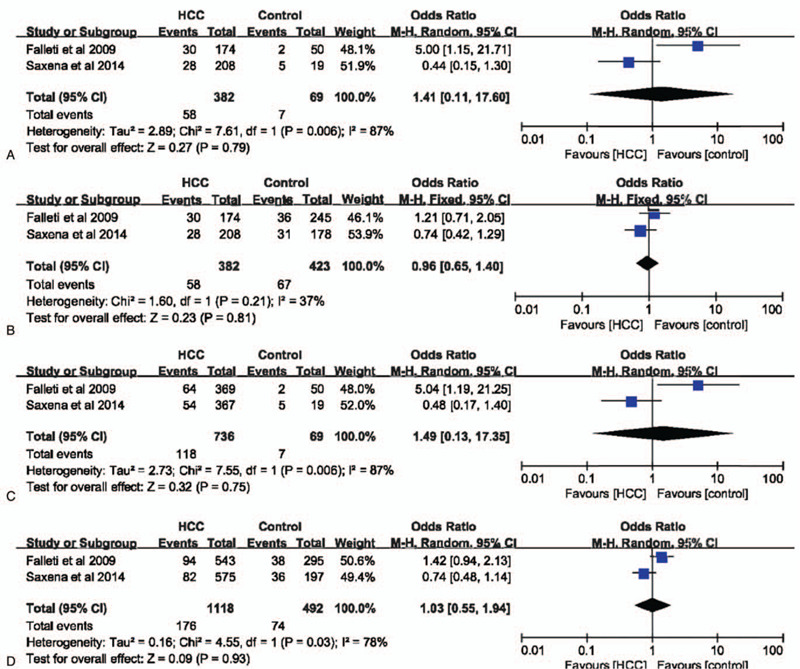
The association between interleukin-6 gene –597G>A polymorphism and hepatocellular carcinoma susceptibility based on overall controls. A. Codominant model; B. Dominant model; C. Recessive model; D. Allele model.

**Figure 9:**
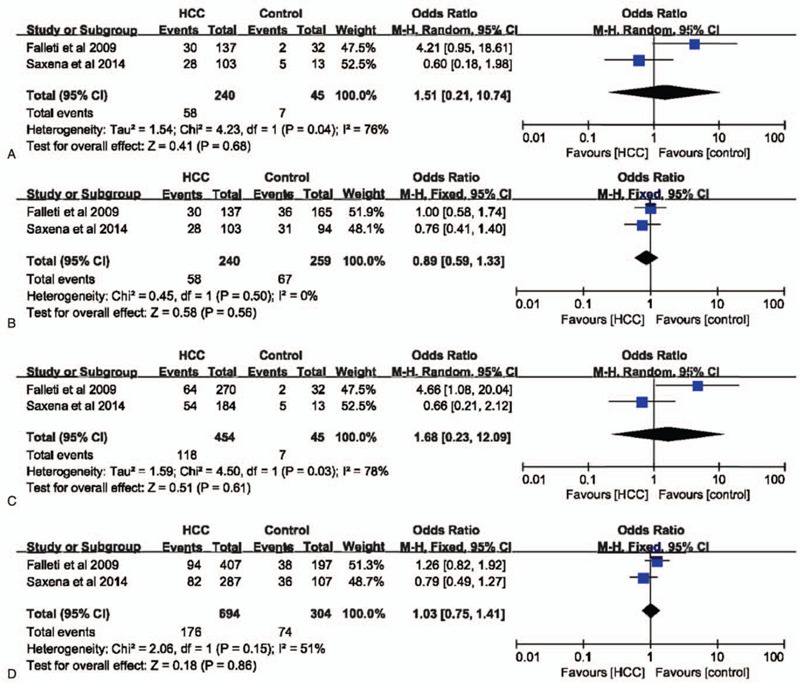
The association between interleukin-6 gene –597G>A polymorphism and hepatocellular carcinoma susceptibility based on normal controls. A. Codominant model; B. Dominant model; C. Recessive model; D. Allele model.

IL-6 –597G>A polymorphism was not significantly related with the risk of HCC in dominant (GG vs AA+GA: OR = 0.95, 95% CI = 0.59–1.54, *P* = .84, I^2^ = 37%), recessive (GG+GA vs AA: OR = 1.49, 95% CI = 0.13–17.35, *P* = .75, I^2^ = 87%), allele (G vs A: OR = 1.03, 95% CI = 0.55–1.94, *P* = .93, I^2^ = 78%), and codominant (GG vs AA: OR = 1.41, 95% CI = 0.11–17.60, *P* = .79, I^2^ = 87%) models (Fig. 8). In subgroup analysis with healthy individuals as controls, IL-6 –597G>A polymorphism had no influence on the risk of HCC in all models (Fig. 9).

### 3.3 Publication bias

Begg and Egger tests were used to evaluate the publication bias for the studies included in this study. The subsequent results showed no significant publication bias in both Begg and Egger test (Table 2).

**Table 2:** Publication bias analysis of the included studies.

	Begg test		Egger test	
	z	Pr > |z|	t	*P*
–174 G>C				
Codominant	0.000	1.000	1.380	.399
Dominant	1.040	0.296	5.750	.110
Recessive	–0.340	1.000	0.310	.783
Allele	1.040	0.296	5.950	.106
–572 G>C				
Codominant	0.300	0.764	0.400	.706
Dominant	0.600	0.548	0.420	.693
Recessive	0.600	0.548	0.340	.748
Allele	0.300	0.764	0.550	.607

## 4 Discussion

Inflammation is closely associated with the development and progression of cancer, and often common pathways could be observed in both disease statuses.^[27,28]^ Therefore, targeting inflammation represents an attractive strategy both for cancer prevention and for cancer prevention and therapy.^[27]^ IL-6, one of the major cytokines in the tumour microenvironment, is an important factor which is found at high concentrations and known to be deregulated in many types of cancer.^[15,29–31]^ A number of studies have attempted to associate IL-6 gene polymorphisms with the susceptibility of HCC, mainly involving –174G>C, –572G>C, and –597G>A polymorphisms within the promoter sequence.^[14,17–26]^ However, the conclusion was controversial. With the increase of publications in recent years, it is possible to perform an up-to-date meta-analysis.

In codominant and recessive models, IL-6 –174G>C polymorphism was associated with HCC susceptibility with almost no heterogeneity, which was consistent with previous report.^[16]^ In dominant and allele models, IL-6 –174G>C polymorphism had no influence on the risk of HCC. However, the heterogeneity was moderate or severe. Hence, we performed a subgroup analysis based on populations. Finally, we found that in non-Italian Caucasian population, IL-6 –174G>C polymorphism was significantly related with the occurrence of HCC in both dominant and allele models without heterogeneity. When healthy individuals were set as control group. Similar findings could be observed. Taken together, these results indicated that IL-6 –174G>C polymorphism was significant related with the susceptibility of HCC, especially in non-Italian Caucasian population.

IL-6 –572G>C polymorphism has been reported to been linked to HCC development. IL-6 (–572) GC genotype shared a negative association with HCC development among HBV carriers.^[17]^ In a study of Han population, the authors found that –572G>C polymorphism of IL-6 gene was associated with susceptibility to HBV-related HCC in a male Chinese participant cohort.^[18]^ Consistent with previous studies,^[16]^ we found no significant association between IL-6 –572G>C polymorphism and risk of HCC in this meta-analysis. Considering many human disorders have potential linkage with genetic background, especially between Caucasian and non-Caucasian populations, we performed a subgroup analysis for –572G>C polymorphism of IL-6 gene and the risk of HCC in Caucasian and non-Caucasian populations. Again, no significant correlation was found. Collectively, these results indicated that IL-6 –572G>C polymorphism did not influence on the occurrence of HCC.

This study also evaluated the potential association between –597G>A polymorphism of IL-6 gene and HCC susceptibility. No significantly positive connection was observed. Only 2 studies were included, and high heterogeneity existed when assessing the correlation between IL-6 –597G>A polymorphism and HCC susceptibility. The interpret of this result should be cautious.

Our study has several strengths. First, this is the largest study to date evaluating the associations between IL-6 gene polymorphism (–174G>C, –572G>C, and –597G>A) and the risk of HCC. Second, NOS scores of included studies indicated that the quality of literatures was relatively high and the heterogeneity was relatively low in data synthesis. Third, participants from different genetic backgrounds made it possible to analyze the relationships in different populations. However, our study also has some limits. First, for IL-6 –597G>A polymorphism, high heterogeneity existed and limited studies were included when performing the data synthesis. As a result, further study is still needed. Second, academic dissertations and conference papers were not included, so there may have been bias in provision of data.

In summary, we performed this up-to-date meta-analysis to evaluate the association between several common IL-6 gene polymorphisms and the susceptibility of HCC. Finally, we found that –174G>C polymorphism of IL-6 gene was associated with risk of HCC, especially in non-Italian Caucasian population. However, –572G>C and –597G>A polymorphisms of IL-6 gene had no impact on the incidence of HCC.

## Acknowledgments

We thank the Department of Hepatology and Institute of Translational Medicine, the First Hospital of Jilin University, for their assistance in this work.

## Author contributions

Pei-Pei An, Li-Na Feng, Xiao-Xue Zhang, and Qing-Long Jin conceived the study. Pei-Pei An, Li-Na Feng, Xiao-Xue Zhang, and Qing-Long Jin designed the study and analyzed the data. Pei-Pei An and Qing-Long Jin wrote this manuscript. All authors discussed and revised the manuscript before submission.

**Conceptualization:** Pei Pei An, Qing-Long Jin.

**Data curation:** Pei Pei An, Li Na Feng, Xiao Xue Zhang, Qing-Long Jin.

**Formal analysis:** Pei Pei An, Li Na Feng, Qing-Long Jin.

**Funding acquisition:** Pei Pei An, Qing-Long Jin.

**Investigation:** Pei Pei An, Li Na Feng, Xiao Xue Zhang, Qing-Long Jin.

**Methodology:** Pei Pei An, Li Na Feng, Xiao Xue Zhang, Qing-Long Jin.

**Project administration:** Pei Pei An, Xiao Xue Zhang, Qing-Long Jin.

**Resources:** Pei Pei An, Li Na Feng, Qing-Long Jin.

**Software:** Pei Pei An, Li Na Feng, Xiao Xue Zhang, Qing-Long Jin.

**Supervision:** Qing-Long Jin.

**Validation:** Pei Pei An, Li Na Feng, Xiao Xue Zhang, Qing-Long Jin.

**Visualization:** Xiao Xue Zhang, Qing-Long Jin.

**Writing – original draft:** Pei Pei An, Qing-Long Jin.

**Writing – review & editing:** Pei Pei An, Qing-Long Jin.

## Supplementary Material

Supplemental Digital Content

## Supplementary Material

Supplemental Digital Content

## Supplementary Material

Supplemental Digital Content

## Supplementary Material

Supplemental Digital Content

## Supplementary Material

Supplemental Digital Content

## References

[1] Villanueva A Hepatocellular carcinoma. N Engl J Med 2019;380:450–1462. 10.1056/NEJMra1713263 30970190

[2] Yang JD Hainaut P Gores GJ A global view of hepatocellular carcinoma: trends, risk, prevention and management. Nat Rev Gastroenterol Hepatol 2019;16:589–604. 31439937 10.1038/s41575-019-0186-y PMC6813818

[3] Elhendawy M Abdul-Baki EA Abd-Elsalam S MicroRNA signature in hepatocellular carcinoma patients: identification of potential markers. Mol Biol Rep 2020;47:4945–53. 32430845 10.1007/s11033-020-05521-4

[4] Du Z Liu X Wei X Quantitative proteomics identifies a plasma multi-protein model for detection of hepatocellular carcinoma. Sci Rep 2020;10:15552. 32968147 10.1038/s41598-020-72510-9 PMC7511324

[5] El-Khoueiry AB Sangro B Yau T Nivolumab in patients with advanced hepatocellular carcinoma (CheckMate 040): an open-label, non-comparative, phase 1/2 dose escalation and expansion trial. Lancet 2017;389:2492–502. 28434648 10.1016/S0140-6736(17)31046-2 PMC7539326

[6] Abdel Ghafar MT Morad MA El-Zamarany EA Autologous dendritic cells pulsed with lysate from an allogeneic hepatic cancer cell line as a treatment for patients with advanced hepatocellular carcinoma: a pilot study. Int Immunopharmacol 2020;82:106375. 32169808 10.1016/j.intimp.2020.106375

[7] Lee JH Lee JH Lim YS Adjuvant immunotherapy with autologous cytokine-induced killer cells for hepatocellular carcinoma. Gastroenterology 2015;148:1383–91. 25747273 10.1053/j.gastro.2015.02.055

[8] Naugler WE Sakurai T Kim S Gender disparity in liver cancer due to sex differences in MyD88-dependent IL-6 production. Science 2007;317:121–4. 17615358 10.1126/science.1140485

[9] El-Sharawy S Negm OE Abd-Elsalam S Study of toll-like receptor 3 gene polymorphism as a novel risk factor for HCV-related hepatocellular carcinoma in Egypt. Curr Cancer Drug Targets 2020;20:382–9. 32189594 10.2174/1568009620666200319102929

[10] Ringelhan M Pfister D O’Connor T The immunology of hepatocellular carcinoma. Nat Immunol 2018;19:222–32. 29379119 10.1038/s41590-018-0044-z

[11] Yang YM Kim SY Seki E Inflammation and liver cancer: molecular mechanisms and therapeutic targets. Semin Liver Dis 2019;39:26–42. 30809789 10.1055/s-0038-1676806 PMC6616367

[12] He G Dhar D Nakagawa H Identification of liver cancer progenitors whose malignant progression depends on autocrine IL-6 signaling. Cell 2013;155:384–96. 24120137 10.1016/j.cell.2013.09.031 PMC4015514

[13] Bergmann J Müller M Baumann N IL-6 trans-signaling is essential for the development of hepatocellular carcinoma in mice. Hepatology 2017;65:89–103. 27770462 10.1002/hep.28874

[14] Giannitrapani L Soresi M Giacalone A IL-6 -174G/C polymorphism and IL-6 serum levels in patients with liver cirrhosis and hepatocellular carcinoma. OMICS 2011;15:183–6. 21329460 10.1089/omi.2010.0093

[15] Aleksandrova K Boeing H Nöthlings U Inflammatory and metabolic biomarkers and risk of liver and biliary tract cancer. Hepatology 2014;60:858–71. 24443059 10.1002/hep.27016 PMC4231978

[16] Liu Y Gao SJ Du BX Association of IL-6 polymorphisms with hepatocellular carcinoma risk: evidences from a meta-analysis. Tumour Biol 2014;35:3551–61. 24318992 10.1007/s13277-013-1469-5

[17] Saxena R Chawla YK Verma I IL-6(-572/-597) polymorphism and expression in HBV disease chronicity in an Indian population. Am J Hum Biol 2014;26:549–55. 24841049 10.1002/ajhb.22562

[18] Tang S Yuan Y He Y Genetic polymorphism of interleukin-6 influences susceptibility to HBV-related hepatocellular carcinoma in a male Chinese Han population. Hum Immunol 2014;75:297–301. 24530755 10.1016/j.humimm.2014.02.006

[19] Badawy AA Othman G Elabbasy LM IL-6 -572G/C and -174G/C polymorphisms association with hepatitis C virus-induced hepatocellular carcinoma. Br J Biomed Sci 2019;76:201–4. 31314698 10.1080/09674845.2019.1642562

[20] Madkour B Gad A Hamdy MS Interleukin-6-572 promoter gene polymorphism and its association with chronic hepatitis C-induced hepatocellular carcinoma: an Egyptian study. Comp Clin Pathol 2018;27:161–5.

[21] Bei CH Bai H Yu HP Combined effects of six cytokine gene polymorphisms and SNP-SNP interactions on hepatocellular carcinoma risk in Southern Guangxi, China. Asian Pac J Cancer Prev 2014;15:6961–7. 25169554 10.7314/apjcp.2014.15.16.6961

[22] Falleti E Fabris C Toniutto P Interleukin-6 polymorphisms and gender: relationship with the occurrence of hepatocellular carcinoma in patients with end-stage liver disease. Oncology 2009;77:304–13. 19940521 10.1159/000260057

[23] Ben-Ari Z Mor E Papo O Cytokine gene polymorphisms in patients infected with hepatitis B virus. Am J Gastroenterol 2003;98:144–50. 12526950 10.1111/j.1572-0241.2003.07179.x

[24] Park BL Lee HS Kim YJ Association between interleukin 6 promoter variants and chronic hepatitis B progression. Exp Mol Med 2003;35:76–82. 12754410 10.1038/emm.2003.11

[25] Migita K Miyazoe S Maeda Y Cytokine gene polymorphisms in Japanese patients with hepatitis B virus infection--association between TGF-beta1 polymorphisms and hepatocellular carcinoma. J Hepatol 2005;42:505–10. 15763337 10.1016/j.jhep.2004.11.026

[26] Ognjanovic S Yuan JM Chaptman AK Genetic polymorphisms in the cytokine genes and risk of hepatocellular carcinoma in low-risk non-Asians of USA. Carcinogenesis 2009;30:758–62. 19126646 10.1093/carcin/bgn286 PMC2675648

[27] Singh N Baby D Rajguru JP Inflammation and cancer. Ann Afr Med 2019;18:121–6. 31417011 10.4103/aam.aam_56_18 PMC6704802

[28] Barabutis N Schally AV Siejka A P53, GHRH, inflammation and cancer. EBioMedicine 2018;37:557–62. 30344124 10.1016/j.ebiom.2018.10.034 PMC6284454

[29] Ogawa H Koyanagi-Aoi M Otani K Interleukin-6 blockade attenuates lung cancer tissue construction integrated by cancer stem cells. Sci Rep 2017;7:12317. 28951614 10.1038/s41598-017-12017-y PMC5615065

[30] Browning L Patel MR Horvath EB IL-6 and ovarian cancer: inflammatory cytokines in promotion of metastasis. Cancer Manag Res 2018;10:6685–93. 30584363 10.2147/CMAR.S179189 PMC6287645

[31] Ham IH Oh HJ Jin H Targeting interleukin-6 as a strategy to overcome stroma-induced resistance to chemotherapy in gastric cancer. Mol Cancer 2019;18:68. 30927911 10.1186/s12943-019-0972-8 PMC6441211

