# Supplementary material for: Association of interleukin-6 gene polymorphisms with the risk of hepatocellular carcinoma: An up-to-date meta-analysis
Source: Medicine (Baltimore). 2020 Dec 11;99(50):e23659. doi: 10.1097/MD.0000000000023659 (PMC7738155; doi:10.1097/MD.0000000000023659)
Supplement: Supplemental Digital Content [file medi-99-e23659-s004.docx]

**Supplemental Figure 4**


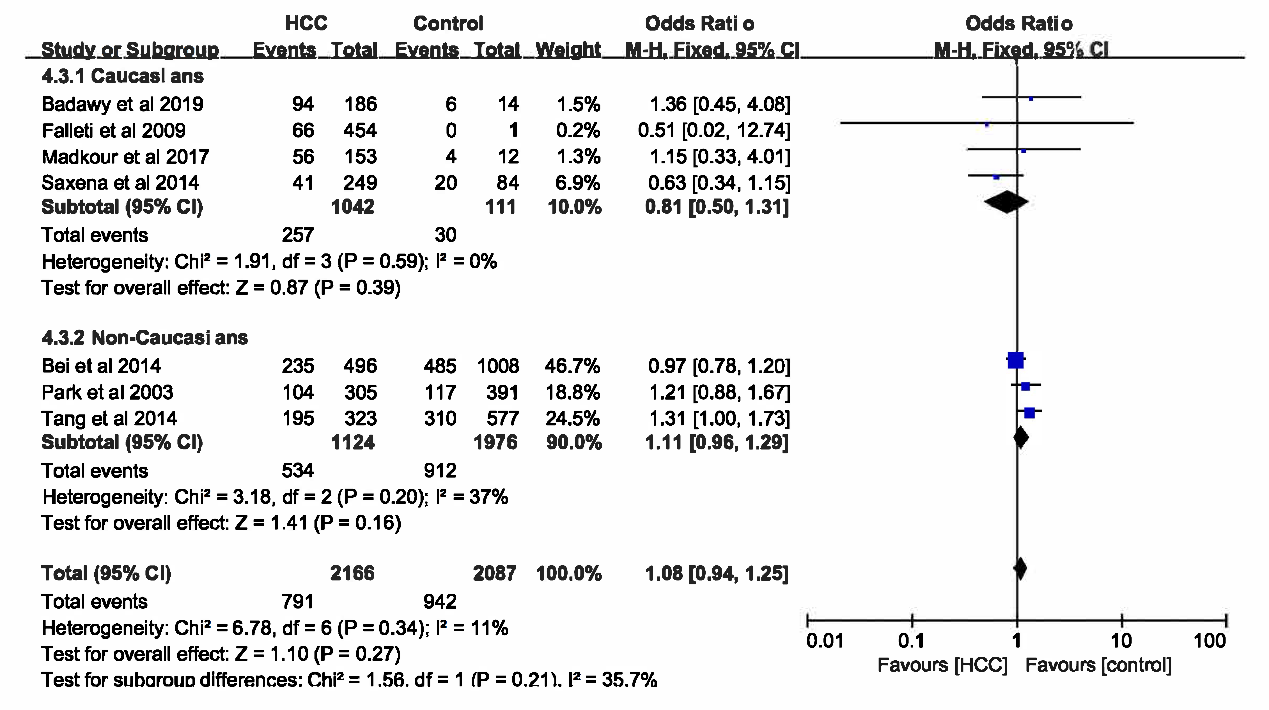


Supplemental Figure 4 The association between IL-6 gene -572G>C polymorphism and HCC susceptibility in Caucasian and non-Caucasian populations based on overall controls in recessive model.
